# High Genetic Diversity of *Enterococcus faecium* and *Enterococcus faecalis* Clinical Isolates by Pulsed-Field Gel Electrophoresis and Multilocus Sequence Typing from a Hospital in Malaysia

**DOI:** 10.1155/2013/938937

**Published:** 2013-05-29

**Authors:** Poh Leng Weng, Ramliza Ramli, Mariana Nor Shamsudin, Yoke-Kqueen Cheah, Rukman Awang Hamat

**Affiliations:** ^1^Department of Medical Microbiology and Parasitology, Faculty of Medicine and Health Sciences, Universiti Putra Malaysia, 43400 Serdang, Malaysia; ^2^Department of Medical Microbiology and Immunology, Universiti Kebangsaan Malaysia Medical Centre, Jalan Yaakob Latif, Bandar Tun Razak, 56000 Kuala Lumpur, Malaysia; ^3^Department of Biomedical Sciences, Faculty of Medicine and Health Sciences, Universiti Putra Malaysia, 43400 Serdang, Malaysia

## Abstract

Little is known on the genetic relatedness and potential dissemination of particular enterococcal clones in Malaysia. We studied the antibiotic susceptibility profiles of *Enterococcus faecium* and *Enterococcus faecalis* and subjected them to pulsed-field gel electrophoresis (PFGE) and multilocus sequence typing (MLST). *E. faecium* and *E. faecalis* displayed 27 and 30 pulsotypes, respectively, and 10 representative *E. faecium* and *E. faecalis* isolates (five each) yielded few different sequence types (STs): ST17 (2 isolates), ST78, ST203, and ST601 for *E. faecium*, and ST6, ST16, ST28, ST179, and ST399 for *E. faecalis*. Resistance to tazobactam-piperacillin and ampicillin amongst *E. faecium* isolates was highly observed as compared to *E. faecalis* isolates. All of the isolates were sensitive to vancomycin and teicoplanin. The presence of epidemic and nosocomial strains of selected *E. faecium* STs: 17, 78, and 203 and *E. faecalis* ST6 as well as high rates of resistance to multiple antibiotics amongst *E. faecium* isolates is of a particular concern.

## 1. Introduction

Enterococci are part of the normal intestinal microflora of most mammals and birds [1] and have emerged as important nosocomial and community pathogens in recent years [2]. Most enterococcal infections in hospitals are due to *Enterococcus faecalis* and *Enterococcus faecium*. Both species are commonly isolated from patients with bacteremia, surgical sites infections, urinary tract infections, and device-related infections [3, 4]. Enterococci create concerns to healthcare practitioners worldwide due to their increasing trend of antimicrobial resistance and great adaptability in hospital environments [5]. Like other nosocomial pathogens, the transmission of enterococci is often associated with the hands of health care workers.

The Center for Diseases Control and Prevention (CDC) has estimated that up to 4 million of hospitalized patients will succumb to hospital-acquired infection each year leading to increased hospital stay and costs [6]. In 2006 and 2007 alone, there were 2263 and 1647 cases of enterococcal infections, respectively, reported in several Malaysian hospitals (unpublished data). However, this data is insufficient to elucidate the current epidemiology of enterococcal infections locally. Hence, this study was designed to characterize the genetic relatedness of enterococcal strains from a tertiary teaching hospital by pulsed-field gel electrophoresis (PFGE) and multilocus sequence typing (MLST).

## 2. Materials and Methods

### 2.1. Bacterial Isolates

Non-repetitive clinical enterococcal isolates were collected from blood, pus, urine, vaginal and sterile body fluid from May 2009 and March 2010 from a tertiary teaching hospital. This hospital is one of the largest referral teaching hospitals in Malaysia with 38 wards and 819 beds. No reported cases of enterococcal outbreaks were reported during the study period.

### 2.2. Microbiological Identification of Vancomycin-Susceptible Enterococci

Enterococci were identified by using conventional biochemical tests [7], Remel RapID Strep Kit (Oxford, UK), and confirmed with species specific PCR previously described by Kariyama et al. [8]. The disk diffusion method was used for antibiotic susceptibility testing and screening for high-level gentamicin resistance, and the results were interpreted according to the Clinical and Laboratory Standards Institute guidelines [9]. Intermediate level of resistance by the disk diffusion method was considered resistant in this study. High-level gentamicin resistance (HLGR) phenotype was defined as resistant to the high content of gentamicin (120 *μ*g/mL) by the disk diffusion method [9] and multidrug resistant strains were defined as strains that are resistant to one or more agents in three or more antimicrobial categories [10].

### 2.3. Molecular Typing of Vancomycin-Susceptible Enterococci

The plugs were lysed in lysis buffer supplemented with RNase (5 mg/mL) and lysozyme (1 mg/mL) which was incubated overnight, followed by fresh lysis buffer with proteinase K (0.5 mg/mL) at 50°C for 48 hours. *Enterococcus* DNA was digested with 20 U of *SmaI* restriction enzyme (Promega, USA) [11]. Electrophoresis was performed in 1% agarose (Seakem Gold, Lonza, USA) on CHEF DRII system (Bio-Rad Laboratories, USA) at 6 V/cm, with linear switching interval ramps from 3.5 s to 25 s for 12 hours at 14°C for the first block, and subsequently followed by 1 s to 5 s for 8 hours for the second block with 0.5X Tris-borate-EDTA [12]. *Salmonella* serotype Braenderup H9812 DNA marker was used for the standard molecular weight and size determinations [13].

The DNA banding patterns were analysed with the use of BioNumerics v. 6.10 software (Applied Maths, Saint-Martens-Latem, Belgium) using Dice coefficient of similarity with band tolerance of 1% and cluster analysis based on the unweighted pair group method with arithmetic averages (UPGMA).

MLST was performed for *E. faecium* according to Homan et al. [14] with primers of the seven housekeeping genes used which are shown in Table 1. Meanwhile, the MLST of *E. faecalis* was performed according to primers and procedures established by Ruiz-Garbajosa et al. [15]. Purified PCR products were then sequenced using commercial sequencing services (First Base Sdn Bhd., Malaysia). MLST sequences were then queried into the MLST databases, that is, (http://efaecium.mlst.net/) and (http://efaecalis.mlst.net/) to determine their sequence types. Unique sequences were submitted to the curator for the assignment of a new allelic profile and sequence type (ST).

## 3. Results

A total of nonrepetitive 59 VSE isolates were analysed of which 31 *E. faecalis* were isolated from pus (17), blood (11), and vaginal (2) and sterile body fluid (1) and 28 *E. faecium* isolates were isolated from pus (8), blood (14), and urine (6) samples.

The rate of resistance to tazobactam-piperacillin, ampicillin, penicillin and high-level gentamicin amongst *E. faecium* isolates was 96.4%, 92.9%, 89.3%, and 82.1%, respectively. Meanwhile, *E. faecalis* isolates exhibited 3.2%, 3.2%, 9.7%, and 38.7% resistance to ampicillin, tazobactam-piperacillin, penicillin and high-level gentamicin, respectively. *E. faecium* exhibited higher multidrug resistant strains as compared to *E. faecalis* (89.1% versus 3.2%). Interestingly, all enterococci isolates were susceptible to vancomycin and teicoplanin.

**Figure 1:**
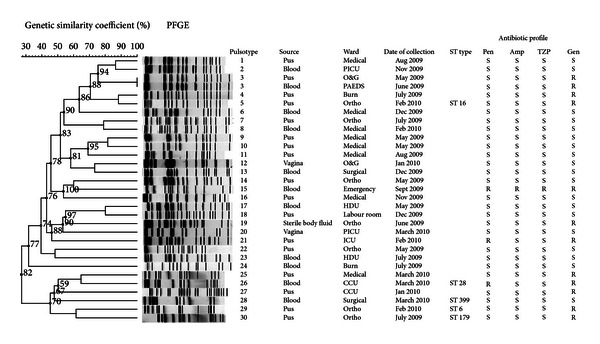
Dendrogram of genetic relatedness among *E. faecalis* strains (BioNumerics 6.10, Applied Maths, Saint-Martens-Latem, Belgium). Pulsotype refers to subtypes labelled as 1, 2, 3, and so forth. Date of collection refers to the date of the isolates that were collected and identified as *E. faecalis*. ST type refers to the assigned sequence type based on MLST. Ortho: Orthopedic ward; PICU: Pediatric Intensive Care Unit; O & G: Obstetrics & Gynecology ward; HDU: High Dependency Unit; CCU: Critical Care Unit; ICU: Intensive Care Unit; S: sensitive and R; resistant; Pen: penicillin, Amp; ampicillin; TZP: tazobactam-piperacillin; Gen: gentamicin (120 *μ*g). No distinct clusters observed.

**Figure 2:**
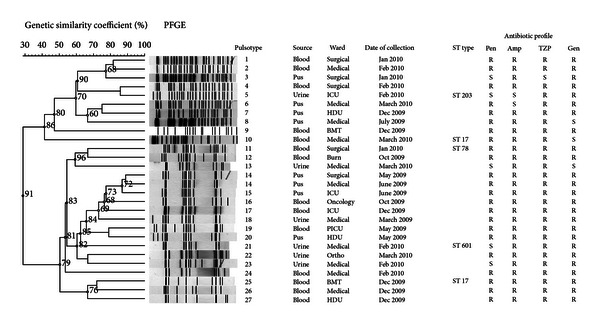
Dendrogram of genetic relatedness among *E. faecium* strains (BioNumerics 6.10, Applied Maths, Saint-Martens-Latem, Belgium). ICU: Intensive Care Unit; HDU: High Dependency Unit; BMT: Bone Marrow Transplant Unit; PICU: Pediatric Intensive Care Unit. Two distinct clusters were observed, cluster I (pulsotype 1–10) and cluster II (pulsotype 11–27).

Genetic relationships between the enterococcal isolates from the study were examined using PFGE analysis with a homology cut-off value of 90%. As shown in Figures 1 and 2, the genetic relatedness of *E. faecalis* (*n* = 31) and *E. faecium* (*n* = 28) revealed 30 and 27 pulsotypes with a low level of homology between strains in both species. However, in *E. faecium*, two distinct clusters were observed. Cluster I and cluster II comprised pulsotype 1 to 10 and pulsotype 11 to 27, respectively. As for pulsotype 14, two identical *E. faecium* isolates were detected with similar antibiogram patterns in two different patients from two different wards at different periods of admission. In addition, pulsotype 3 was identically detected in two *E. faecalis* strains with similar antibiogram patterns isolated in two different patients. Further information revealed that these isolates were also different in terms of the period of isolation, the location of the ward, and the type of sample.

MLST was performed for only five isolates of each species to determine the STs due to its high cost and labor intensive. The isolates were selected based on the antibiotic and PFGE profiles. ST types of *E. faecalis* were identified as follows: ST6, ST16, ST28, ST179, and ST399, whereas *E. faecium* isolates revealed ST17 (2 strains), ST78, ST203, and ST601.

## 4. Discussion

In general, *E. faecium* isolates exhibited high resistance rates to antibiotics compared to *E. faecalis* in our study. For instance, 92.9% of them were resistant to ampicillin. In contrast, resistance to ampicillin was only observed in 3.2% of *E. faecalis* isolates. Resistance to ampicillin is very common among *E. faecium* clinical isolates as reported by several studies [7, 16, 17]. For example, Miskeen and Deodhar [18] used the disc diffusion method and demonstrated 75.0% and 17.0% of 26 *E. faecium* and 128 *E. faecalis* isolates, respectively, and exhibited resistance to ampicillin. This is not surprising as the decreased affinity of penicillin-binding proteins or plasmid-mediated *β*-lactamases might be responsible for this resistance mechanism and *E. faecium* has a great ability to acquire resistant determinants [19].

Treatment for serious enterococcal infections requires the combination of an aminoglycoside with *β*-lactams drugs such as penicillin/ampicillin for a synergistic bactericidal effect. However, enterococci strains that show a high-level aminoglycoside resistance (HLAR) phenotype would no longer be susceptible to aminoglycosides and could not be used for the combination therapy [20]. Since most of the enterococcal infections utilizing gentamicin for their synergism [21], screening for a HLAR gentamicin is usually acceptable in most diagnostic laboratories. In our study, 82.1% *E. faecium* exhibited resistance to high-level gentamicin, whereas 38.7% of *E. faecalis* were resistant. Similar to our study, Kacmaz and Aksoy [22] demonstrated 88.0% (22/25) and 16.4% (34/207) of *E. faecium* and *E. faecalis*, respectively, and were resistant to high-level gentamicin. Few reports of the isolation of HLGR *E. faecium* have been documented in several other countries [23, 24]. The most worrying part is that this resistance determinant is transferable among bacteria via plasmids [25]. Surprisingly, no vancomycin and teicoplanin resistance was detected in our study. The prevalence of vancomycin-resistant enterococci (VRE) in Malaysia is very low, and only a few sporadic cases of vancomycin-resistant enterococci (VRE) isolates have been reported so far [26, 27].

*E. faecalis* and *E. faecium *clinical isolates demonstrated a high level of diversity by PFGE typing during the study period. Similar findings from earlier studies demonstrated a high genetic diversity amongst these isolates originating from the same and/or different hospitals [28, 29]. For instance, D'Azevedo et al. [29] studied 455 clinical enterococcal isolates in five different hospitals and found the genetic diversity ranging from low (60.0%) to high similarity (95.0%). In our study, the PFGE patterns exhibited high heterogeneity amongst strains although the recovery of pulsotype 3 and 14 (Figures 1 and 2) in different patients from different wards with similar antibiogram profiles might probably show the possibility of cross-transmission of strains across wards within the hospital. PFGE has been considered as the “gold standard” for the study of hospital outbreaks because of its high degree of isolate differentiation [30]. However, MLST has emerged as an important tool to study the long-term epidemiology and the population structure and patterns of evolutionary descent [31].

MLST analysis of five selected *E. faecalis* strains revealed several STs such as ST6, ST16, ST28, ST179, and ST399 in our study. The presence of ST6 in this study, which is associated with clonal-complex 2 (CC2), deserves special attention as CC2 is commonly reported amongst nosocomial isolates and represents hospital-adapted complexes [32]. Moreover, CC2 is linked to vancomycin susceptibility with lower incidence of enterococcal surface protein (*esp*) gene carriage but exhibits high-level resistance to aminoglycosides [16]. ST6 in our study was sensitive to vancomycin but resistant to HLGR. ST 28 of CC87 has also been regarded as high-risk CCs similar to CC2 [15].

MLST analysis for *E. faecium* yielded ST17, ST78 and ST203 which are derived from CC17. CC17 is a major group of genetic lineage of *E. faecium* that has widely spread worldwide [3] and it is associated with hospital outbreaks [33, 34]. Two newly discovered STs were obtained at the time of database query that is, ST 399 (*E. faecalis*) and ST 601 (*E. faecium*). Nonetheless, the significance of these STs is yet uncertain.

Our study has several limitations. Because of the limited number of isolates, it is very difficult to draw firm conclusions particularly on the distribution of STs. However, resistance rates to important antibiotics as observed among *E. faecium* isolates cannot be ignored as horizontal transfer of resistance and virulence determinants is imminent among enterococci.

## 5. Conclusion

The high genetic variability amongst enterococci isolates in this study provides some information on the local dissemination and genetic relatedness, as well as the antibiotic patterns of our enterococcal isolates. Although little information can be deduced from the findings of their sequence types, constant monitoring and active surveillance of enterococcal infections should always be emphasized in this hospital.

## Figures and Tables

**Table 1:** List of *E. faecium* primers used in the study.

Housekeeping genes	Primer sequences (5′-3′)
*adk *	Forward GAACCTCATTTTAATGGGG Reverse TGATGTTGATAGCCAGACG
*atpA *	Forward CGG TTC ATA CGG AAT GGC ACA Reverse AAG TTC ACG ATA AGC CAC GG
*ddl *	Forward GAG ACA TTG AAT ATG CCT TAT G Reverse AAA AAG AAA TCG CAC CG
*gyd *	Forward CAA ACT GCT TAG CTC CAA GG C Reverse CAT TTC GTT GTC ATA CCA AGC
*gdh *	Forward GGC GCA CTA AAA GAT ATG GT Reverse CCA AGA TTG GGC AAC TTC GTC CCA
*purK *	Forward CAGATTGGCACATTGAAAG Reverse TTCATTCACATATAGCCCG
*pstS *	Forward TTG AGC CAA GTC GAA GCT GGA Reverse CGT GAT CAC GTT CTA CTT CC

*Adk*: adenylate kinase; *atpA*: ATP synthase, alpha subunit; *ddl*: D-alanine: D-alanine ligase; *gyd*: glyceraldehyde-3-phosphate dehydrogenase; *gdh*: glucose-6-phosphate dehydrogenase; *purK*: phosphoribosylaminoimidazole carboxylase ATPase subunit; *pstS*: phosphate ATP-binding cassette transporter.
